# Digitalization and job stress: exploring the mediating roles of job and personal aversion risk with gender as a moderator

**DOI:** 10.3389/fpsyg.2024.1370711

**Published:** 2024-06-26

**Authors:** Shibo Han

**Affiliations:** Faculty of Business, City University, Kuala Lumpur, Malaysia

**Keywords:** digitalization, job stress, risk aversion, gender differences, time-lagged study, ICT professionals

## Abstract

**Background:**

This time-lagged study delves into the impact of digitalization on job stress. Digitalization is defined as the incorporation of digital technologies into various aspects of work life, fundamentally transforming processes, interactions, and decision-making.

**Aim:**

The present research focuses on the mediating roles of job-related risk and personal risk, and the moderating effect of gender. We hypothesized that employees’ aversion to risks, both in their professional and personal facets, mediates the relationship between the rapid digitalization of their work environment and the resultant job stress. Regarding gender as a moderator, recent research suggests that gender can influence the experience of workplace stress, with women often experiencing higher levels of stress than men in certain situations. This indicates that gender might also moderate the relationship between digital living, risk perception, and job-related stress. This approach allows for an examination of the ways in which digital technology adoption influences workplace stress, considering the temporally spaced data.

**Methods:**

Conducted over three waves of data collection among 795 Chinese employees, the research utilizes Hayes’s Model 8, adept at revealing the dynamics of digitalization’s influence in the workplace and its effects on individual well-being.

**Results:**

The study corroborates Hypothesis 1 by establishing a significant, albeit less pronounced, relationship between digital living and job stress. The findings also support Hypothesis 2 by demonstrating that both job risk and personal risk mediate this relationship. The study’s results also validate Hypothesis 3, indicating that gender moderates the relationship between digital living, job risk, personal risk, and job stress. Finally, the significant interaction effects found in the study, particularly the stronger conditional negative effect of digital living on perceptions of job and personal risks for males, despite the absence of statistical significance failed to support Hypothesis 4.

**Implications:**

This study sheds light on the dynamics of job stress in the context of a digitalizing work environment. The results have important implications for designing workplace strategies and interventions that are sensitive to risk perceptions and gender differences in the digital era.

## Introduction

In the contemporary digital era, Information and Communication Technologies (ICT) have become a cornerstone in various sectors, including education, business, and healthcare. These technologies encompass a wide array of tools and resources used for communication, information creation, dissemination, storage, and management, extending from digital devices like computers and smartphones to internet-based technologies, broadcasting mediums, and telephony. Van Dijk (2020) notes that the pervasiveness of ICT not only influences professional domains but also extends to personal and social aspects of life, shaping how individuals interact, learn, and access information.

However, this integration of ICT into daily life is not without its disparities, especially when viewed through the lens of gender. Gender differences in the usage, competencies, and attitudes toward ICT are well-documented (Hargittai and Shafer, 2006; Aydin et al., 2023). Specifically, risk attitudes toward ICT also vary significantly between genders. Job-related risks, focusing on data security and privacy breaches, and personal risks, concerning online privacy and mental health impacts, are perceived differently based on gender (Lee et al., 2014; Karimikia et al., 2020). Females typically exhibit higher risk aversion, particularly regarding online privacy and data security (Gustafsod, 1998). Addressing these differences is vital for promoting female participation in the digital world (Maxfield et al., 2010). Despite ongoing efforts to close the gender gap in the technology industry, persistent inequities remain (Sharma et al., 2021; Qazi et al., 2022; Liao et al., 2023).

Digital living, or the habitual use of ICT in daily life (Gellerstedt et al., 2018), has direct implications for job-related stress. Recent studies highlighted how the use of digital tools, especially during the COVID-19 lockdown, impacted teleworkers’ job stress (Martin et al., 2022) This research suggests that digital tools can enhance job productivity, but their excessive use can lead to information overload, increasing employees’ job stress. At the same time, the existing literature suggests that gender may moderate the relationship between digital living, risk perception, and job-related stress, indicating differing experiences of workplace stress among men and women (Artz et al., 2022; Carmassi et al., 2022; Rodríguez-Modroño, 2022). This study aims to examine the influence of gender on the use of ICT, perceptions of risk, and the job-related stress. By investigating gender as a key variable in how digital tools impact professional stress levels, this research seeks to fill a gap in the current understanding of digital living’s effects in the workplace. The findings are expected to provide evidence-based insights into gender differences in ICT engagement and their implications for occupational well-being. Such knowledge could inform targeted strategies to alleviate job stress and promote gender equality in the digital sphere, with significant implications for policy and organizational practices.

## Literature review

### A gender perspective on information and communication technologies use

The concept of information and communication technologies (ICT) encompasses a diverse range of tools and resources employed for communication, creation, dissemination, storage, and management of information. This broad spectrum includes digital devices such as computers and smartphones, as well as internet-based technologies, broadcasting mediums like radio and television, and telephony. In today’s digital landscape, ICT has become integral to various sectors including education, business, and healthcare, impacting not only professional but also personal and social spheres (van Dijk, 2020). The positive impacts of digitalization on efficiency and performance have been stated from different studies, both at the individual (Deng et al., 2023), group (Zhao et al., 2023) and organizational levels (Ding et al., 2023). Its pervasive influence shapes how individuals interact, learn, and access information, making it a crucial element of modern life.

Gender disparities toward ICT are still supported by empirical research (Enock et al., 2024). Men often engage more with ICT in work-related contexts and are more confident in their technical skills, whereas women typically demonstrate a cautious approach to ICT (Sharma et al., 2021; Qazi et al., 2022). For instance, a study by Hargittai and Shafer (2006) found that men are more likely to engage with ICT in a professional context and exhibit higher confidence in their abilities. Conversely, women often demonstrate a more cautious approach to ICT, influenced by societal norms and expectations that historically positioned technology as a male-dominated field. This cautiousness can impact both personal and professional engagement with technology. Further research (Hupfer et al., 2021) highlighted that women, despite increasing usage, often have less access to ICT resources and face barriers in acquiring technical competencies. These gender-based disparities in ICT engagement impact both personal and professional outcomes (Bhandari, 2019), specifically in developing countries (Rashid, 2016). Some studies found that women tend to perceive ICT as less integral to their personal and professional advancement compared to men (Hupfer et al., 2021). Other studies stated that, when women use the ICT technologies, they tend to show differential using patterns (Zainudeen et al., 2010). Such attitudes not only affect women’s engagement with ICT but also their participation in related educational and career opportunities.

The significance of understanding these gender-based differences in ICT use cannot be overstated. Recognizing and addressing these disparities is key to promoting equitable access and usage of ICT. In the workplace, this understanding can inform policies and practices that encourage female participation in ICT roles and support their career advancement. This is particularly important in industries where gender imbalance is pronounced. In educational contexts, a gender-sensitive approach to ICT education can ensure that both boys and girls are equally prepared and encouraged to engage with technology from a young age (Qazi et al., 2022). Such initiatives are crucial for bridging the gender gap in ICT, fostering a more inclusive and diverse digital environment. As argued by Van Deursen and van Dijk (2019), equal access to and proficiency in ICT is essential for full participation in modern society, making gender inclusivity not only a matter of fairness but also a prerequisite for societal progress.

In conclusion, the literature review reveals that while progress has been made in understanding gender dynamics in ICT, significant work remains in translating this knowledge into action. By addressing these disparities, society can move toward a more equitable and inclusive digital future, where gender does not determine one’s ability to access, use, and benefit from the transformative power of ICT.

### Risk perception about ICT use and gender

In the realm of ICT, the perception of risk is complex, involving both job-related and personal aspects. Job-related risks typically focus on data security, privacy breaches, and the potential for job automation, as highlighted by Karimikia et al. (2020). Personal risks, conversely, often relate to online privacy, cyberbullying, and the impact of digital technology on mental health. Some studies note that these risks are perceived differently based on the user’s role and interaction with ICT, with professionals often prioritizing data security and job automation, and everyday users more concerned with privacy and psychological well-being (Lee et al., 2014).

Gender significantly influences risk perception in ICT. Females typically exhibit higher risk aversion than males, often showing greater concern about online privacy and data security (Gustafsod, 1998). This heightened risk aversion among women is thought to stem from broader social and psychological factors, including societal norms and gendered experiences. Addressing these differences is crucial for reducing gender disparities in ICT, as doing so can promote greater female participation in the digital world (Maxfield et al., 2010).

Despite the acknowledgment of these gender differences in ICT use and risk perception over the past two decades and evolving gender dynamics in the workplace, recent research indicate an ongoing inequity (Sharma et al., 2021; Qazi et al., 2022). The technology industry’s efforts to close the gender gap, as reported (Christensen, 2023), have resulted in only a marginal increase in female representation. Furthermore, the World Economic Forum has called for systemic changes to address these persistent disparities, emphasizing hiring based on skills, a commitment to equal representation, and tracking representation percentages.

Understanding the gendered influences of risk perception in ICT is essential for achieving gender equity in this field. Developing inclusive and equitable ICT policies and practices that address women’s specific concerns can lead to safer online environments and more targeted education and training. By considering the diverse needs and preferences of all users in the design of ICT tools and platforms, we can work toward a digital landscape where gender does not limit full and safe participation in the ICT domain.

### Digital living and job related stress: direct, mediated and moderated relationships

Digital living, defined as being accustomed to using ICT in daily life (Gellerstedt et al., 2018), has become increasingly prevalent in modern society. However, this integration of digital technologies into everyday routines has direct implications for job-related stress.

A recent study (Martin et al., 2022) highlights how the use of digital tools, especially during the COVID-19 lockdown, impacted teleworkers’ job satisfaction and stress. The study found that while a balanced use of digital tools could enhance job productivity, an increased frequency and intensity of use often led to information overload, thereby increasing job stress and reducing job satisfaction and productivity.

Research in stress psychology indicates that individuals who view work-related challenges as threatening are more prone to experiencing stress, alongside symptoms of depression and physical health issues (Bakker et al., 2023). Such individuals might struggle with concentration, reduced problem-solving capabilities, emotional fluctuations, disturbances in sleep, muscle aches, and feelings of lightheadedness. Consequently, these individuals often undergo a depletion of their resources, complicating their ability to navigate the ups and downs of job requirements, which can lead to elevated stress levels (Elshaer et al., 2024). This depletion of energy makes it increasingly challenging to invest effort into work-related tasks. Therefore, individuals with heightened perceptions of risk may find themselves depleted of the necessary energetic and cognitive capacities to effectively manage their work demands, resulting in increased job stress (Ingusci et al., 2021). Additionally, a high risk perception can divert attentional resources, leading to more frequent lapses in focus and inconsistencies in performance on tasks requiring attention. Conversely, employees who interpret their work demands more positively are likely to possess the requisite energetic and cognitive resources to successfully handle intensive workloads and demanding tasks, thereby shielding themselves from the adverse effects of job-related stress (Cho et al., 2020). The mediation effect might involve how individuals perceive and manage risks associated with digital technologies, which in turn influences their experience of stress in the workplace (Gadeyne et al., 2018).

Regarding gender as a moderator, recent research suggests that gender can influence the experience of workplace stress, with women often experiencing higher levels of stress than men in certain situations. This indicates that gender might also moderate the relationship between digital living, risk perception, and job-related stress (Artz et al., 2022). Psychological research highlighted that perceived stress significantly differ as a function of gender, with women showing more than double than men in the high perceived stress values group. This values has been tested among Italian workers searching for employment (Costa et al., 2021), as well as among university professors (Redondo-Flórez et al., 2020), among others. Despite this fact, other studies reported that, at least in some professional fields, gender has been found a moderator, but the group that showed higher values of job stress was males, perhaps given that the unbalanced gender representation in this specific professional field (Lee and Cho, 2016). Related to this moderation hypothesis would be the gender-specific variation in teleworking frequencies, both before and after the COVID-19 pandemic. Studies based on the European Working conditions survey with more than 16.000 participants stated that the under-representation of women among teleworkers remain also when occupational gender segregation is taken into account (Kley and Reimer, 2023). In a similar vein, studies using nationally representative surveys from the UK, showed that among men, teleworkers tend to have higher levels of enjoyment at work, but this is not the case for women (Lu and Zhuang, 2023). Additionally, a study on the effects of working from home on workers’ health notes gender-specific differences, with varying optimal tele-working frequencies for men and women, highlighting the gendered nature of digital living’s impact on health and, by extension, job-related stress (Ellinas et al., 2022).

### Hypotheses

**Figure 1:**
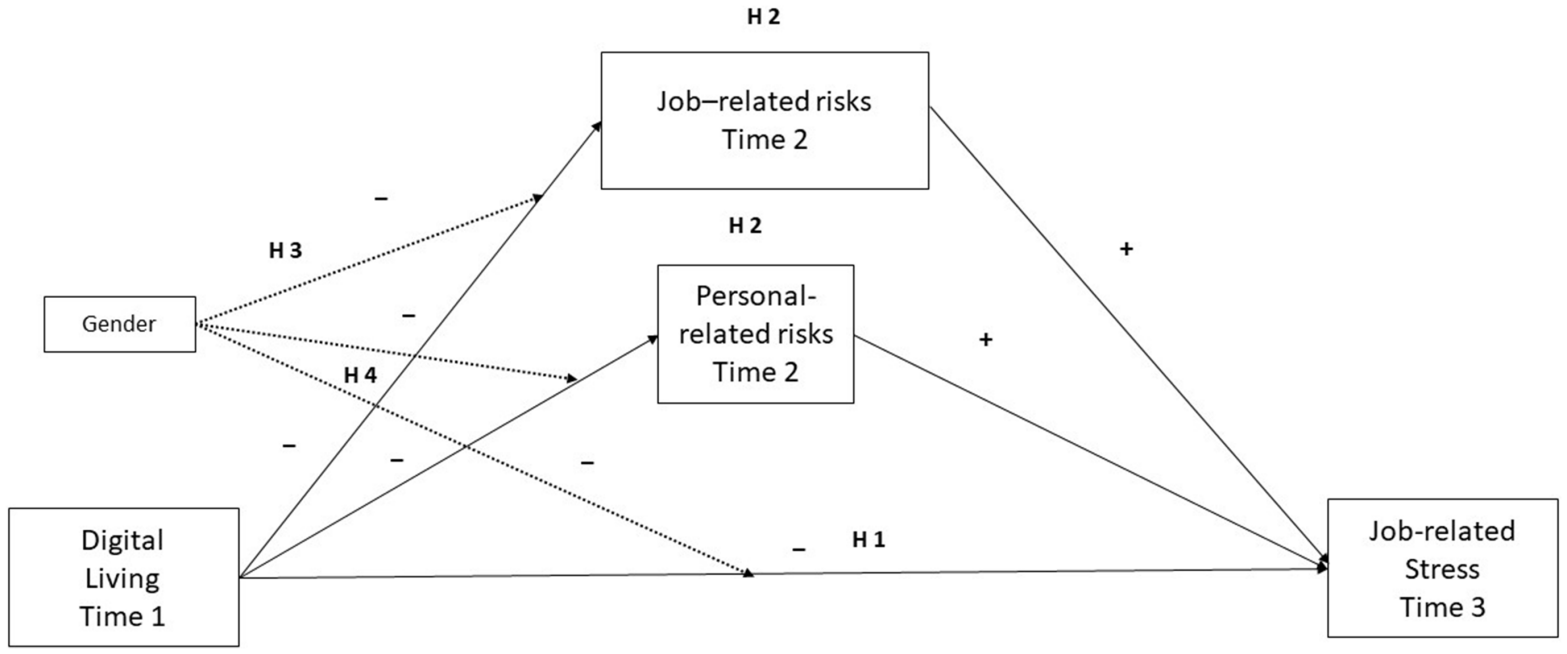
Conceptual diagram. Conditional indirect effect of X on Y through M = (*a*_1i_ + *a*_3i_ *W*) *b*_i_. Conditional direct effect of X on Y = *c*_1_’ + *c*_3_’*W.* Moderation effects are shown with black dotted lines. Gender assessed as 1 = males, 2 = females.

**Figure 2:**
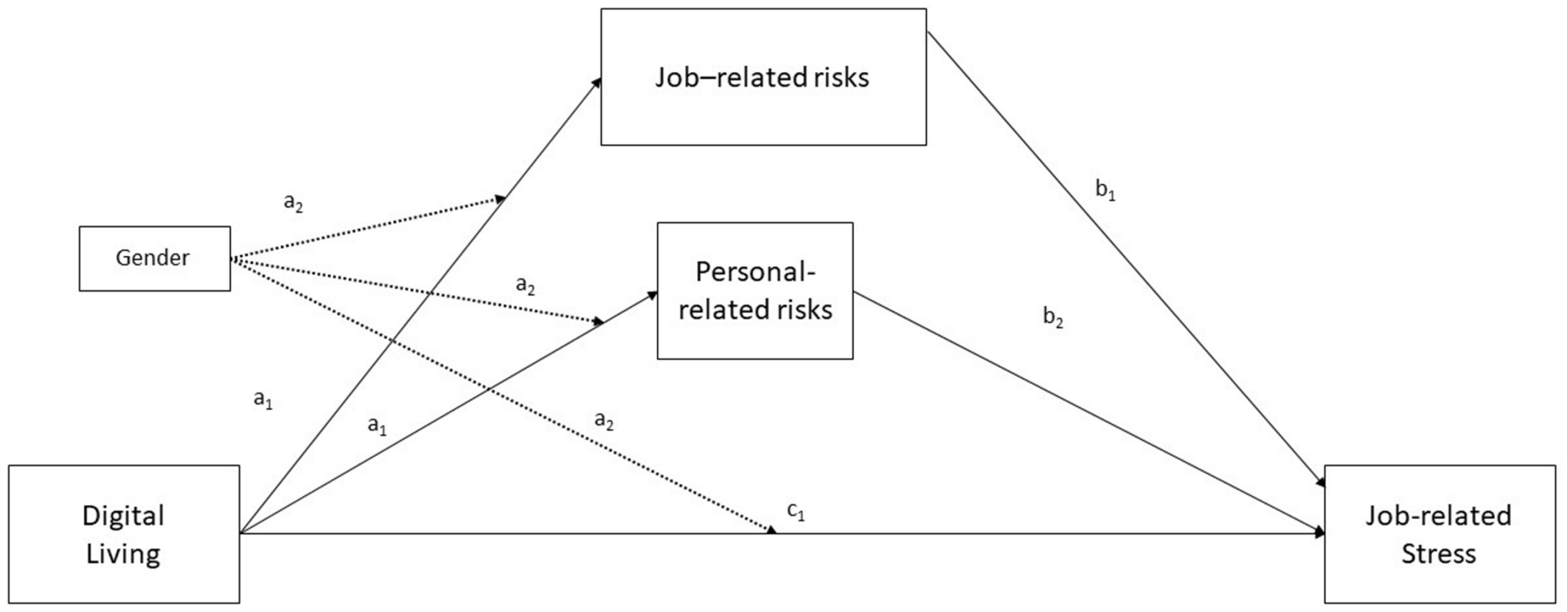
Statistical diagram. Moderation effects are shown with black dotted lines. Conditional indirect effect of *X* on *Y* through *Mi* = (*a* 1*i* + *a* 3*iW*) (*b* 1*i* + *b* 3*iW*). Direct effect of X on Y = c_1_.

Based on the literature, we propose the following conceptual and statistical models (see Figures 1, 2).

Hence, the hypotheses for the present study are exposed as follows:

*H1*: Digital living (Time 1) will negatively impact on job-related stress (Time 3).

*H2*: Perceived risks (Time 2), both Job-related and personal-related, will mediate the relationships between Digital living and job-related stress.

*H3*: Gender (Time 1) will moderate the relationships between Digital living and Perceived risks, both Job-related and personal-related.

*H4*: Gender (Time 1) will moderate the relationships between Digital living and job-related stress.

## Methods

### Instruments

#### Digital living

At Time 1, this variable has been assessed with the digital Living Scale (Gellerstedt et al., 2018), that consists in eighth items. Examples of the items were the following: *Digital technology is an important part of my social life*, and *I would say that digital technology actually helps me a lot in my everyday life.* The scale showed adequate reliability values in the present study (Cronbach’s Alpha = 0.93).

#### Job – related risks

At Time 2, this variable was assessed with three subscales adapted to the ICT’s use at work, that measured: Time-risk (Risks related to time loss during ICT’s use); Privacy (Risks related to concerns regarding loss of personal information when ICT’s use at work), and Security-risks (Risks related to the credibility of the other parts in the interaction or online platform). Three items for each dimension were adapted from Featherman and Pavlou (2003). Examples of items were: *I am afraid it will take a long time to fix errors that occurred online or to get help from ICT services when use ICT at work* (Time-risk), *I am afraid that my personal information will be used for reasons other than commercial activities* (Privacy Risks), and *I am afraid that individual information (usernames, passwords, address, identification details,* etc.*) will be obtained by third parties during ICT’s use at work* (Security Risks). The scale showed adequate reliability values in the present study (Cronbach’s Alpha = 0.90).

#### Personal-related risks

This variable was assessed at Time 2, considering two types of information. The subscales were focused on Social Risk (those related to potential loss of one’s social status resulting from inadequate ICT’s use at work) and Psychological Risks (those related to mental stress and dissatisfaction resulting from a potential failure of ICT’s use at work). Items have been adapted from Featherman and Pavlou (2003) and Ko et al. (2004), including two items the first, and three items the second. The scale showed adequate reliability values in the present study (Cronbach’s Alpha = 0.92).

#### Job–related stress

At Time 3, the *Perceived Stress Scale* has been used for assessing this variable (Cohen et al., 1994). The Perceived Stress Scale is the most widely used tool for measuring stress and it consist in 10 items that are focused in stress as a general phenomenon. In the present study we adapted the redaction of some items to focus more on stress at work. Examples of items were: *In the last month, how often have you been angered because of things (at work) that happened that were outside of your control?* The scale has been validated for Chinese populations of different occupational sectors (Jiang et al., 2023; Xiao et al., 2023), and showed adequate psychometric properties (Du et al., 2023). In the present study, the scale showed adequate reliability value (Cronbach’s Alpha = 0.85).

For all the scales, the answers were in a five points Likert-type scale ranging from 1 (Totally disagree or Never) and 5 (Totally agree or Very often). The scales have been presented to the participants in English. Given that the participants were technology-friendly users, all of them were fluent in English as employees at the China’s ICT sector.

### Participants and procedure

Data were gathered at three distinct intervals over a three-month period in 2023 from individuals employed in China’s ICT sector. The initial release commenced on February 15th, precisely 1 week subsequent to the conclusion of the Chinese New Year celebrations in 2023. The strategy of implementing one measure per month was predicated on the logic of segregating the assessments. This approach was intended to mitigate the risk of common method bias by evaluating the variables at distinct intervals. Such temporal separation ensures that the potential for memory decay and respondent disengagement is minimized, thereby preserving the integrity of the data collected. Spreading out the measurement of various constructs was also beneficial in shortening the duration of each questionnaire. Master’s students at a university in China were involved in the study, receiving practical academic credits for their participation. These students were tasked with enlisting professionals from the ICT sector, resulting in a diverse sample drawn from their personal and professional networks. The research team provided the students with an email template to invite potential participants, which included a link to the consent form and the survey. Participants who completed the initial survey were subsequently provided links for the later data collection phases. In the first phase, Time 1 (T1), participants shared demographic data and information about their digital lifestyle. The second phase Time 2, (T2) focused on collecting data regarding job-related and personal risks. In the final phase Time 3, (T3), participants reported on their stress levels related to their employment.

In this research, 795 Chinese ICT professionals participated. The demographic composition of the sample predominantly consisted of females (57.11%) with an average age of 36.79 years (standard deviation = 9.31). The educational background of the majority (35%) included a college, university, or technical college degree. Detailed sample characteristics, such as contract type and industry sector, can be found in Table 1. The initial survey attracted 1,323 participants. However, the number of respondents varied at different stages of the study (T1: 1323; T2: 950; T3: 801), indicating a gradual decrease in participation. The focus of our analysis is on 795 respondents who consistently provided comprehensive data on both the main and control variables throughout all phases. The Ethics Committee of City University sanctioned the study, adhering to the principles of the Declaration of Helsinki. Researchers fully briefed the participants about the study’s aim and procedures, assuring them of their right to discontinue participation at any point. Informed consent was obtained in writing (via agreement to three preliminary questions on the survey’s opening webpage), and all collected data were anonymized to protect the privacy of the participants.

**Table 1:** Descriptive statistics and Pearson’s correlation matrix (*N* = 795).

Variable	Mean	SD	1	2	3
1. Digital living (T1)	1.115	1.115	—		
2. Job-related risks (T2)	1.209	1.209	−0.473**	—	
3. Personal-related risks (T2)	0.918	0.918	−0.311**	0.439**	—
4. Job-related stress (T3)	0.756	0.756	−0.436**	0.318**	0.313**

**p < 0.001.

### Statistical analyses

The Model 8 (Hayes, 2022) examined the relationships between (X) Digital Living (T1) and (Y) Job-Related Stress (T3), with the mediating roles of (M1) Job-Related Risks (T2) and (M2) Personal-Related Risks (T2), and Gender (T1) as a moderating variable.

Both the mediation hypotheses and the moderated mediation hypotheses are supported when zero is not included in the 95% bias-corrected confidence interval, and it may be concluded that the parameter is significantly different from zero at *p* < 0.05. Moreover, regarding moderated mediation, it was expected that the mediation process varies in line with the different values taken by the moderating variable. This procedure was based on 5,000 bootstrap re-samples and provided a moderated mediation index, as well as estimates of the indirect effect and associated confidence intervals conditional on the specific levels of the moderator, categorized as 1 = males, 2 = females.

## Results

### Mediational analyses

#### Job risk as an outcome variable

The relationship between digital living and job risk was significant. The model summary revealed an R-squared value of 0.2302, suggesting that digital living explained more than 20% of the variance in job risk. The analysis showed a negative coefficient for digital living, indicating that higher engagement in digital living was associated with lower job risk. Gender, included as a moderator, significantly impact this relationship. The interaction effect (Digital Living x Gender) was also found to be significant. The conditional negative effect of Digital living on perception of job risks is stronger for males than for females, supporting Hypothesis 3.

#### Personal risk as an outcome variable

The analysis of personal risk as an outcome variable yielded similar results. The model indicated a significant negative effect of digital living on personal risk, with an R-squared value of 0.1170. Gender played a more pronounced role in this relationship. The interaction term (Digital Living × Gender) was significant, indicating that the impact of digital living on personal risk varied based on gender. The conditional negative effect of Digital living on perception of personal risks is stronger for males than for females, supporting Hypothesis 3. The moderated mediation index showed its statistical significance for both the indirect effect through Job-risks and Personal-related risks.

#### Job-related stress as an outcome variable

In examining job stress, the study found a less pronounced but still significant relationship with digital living, supporting Hypothesis 1. Both job risk and personal risk were included as mediators in this relationship, supporting Hypothesis 2. The indirect effects analysis revealed that digital living influenced job stress through these mediators. The direct effect was not moderated by gender, with different indirect effects observed for males and females, but without statistical significance, failing to support Hypothesis 4.

**Figure 3:**
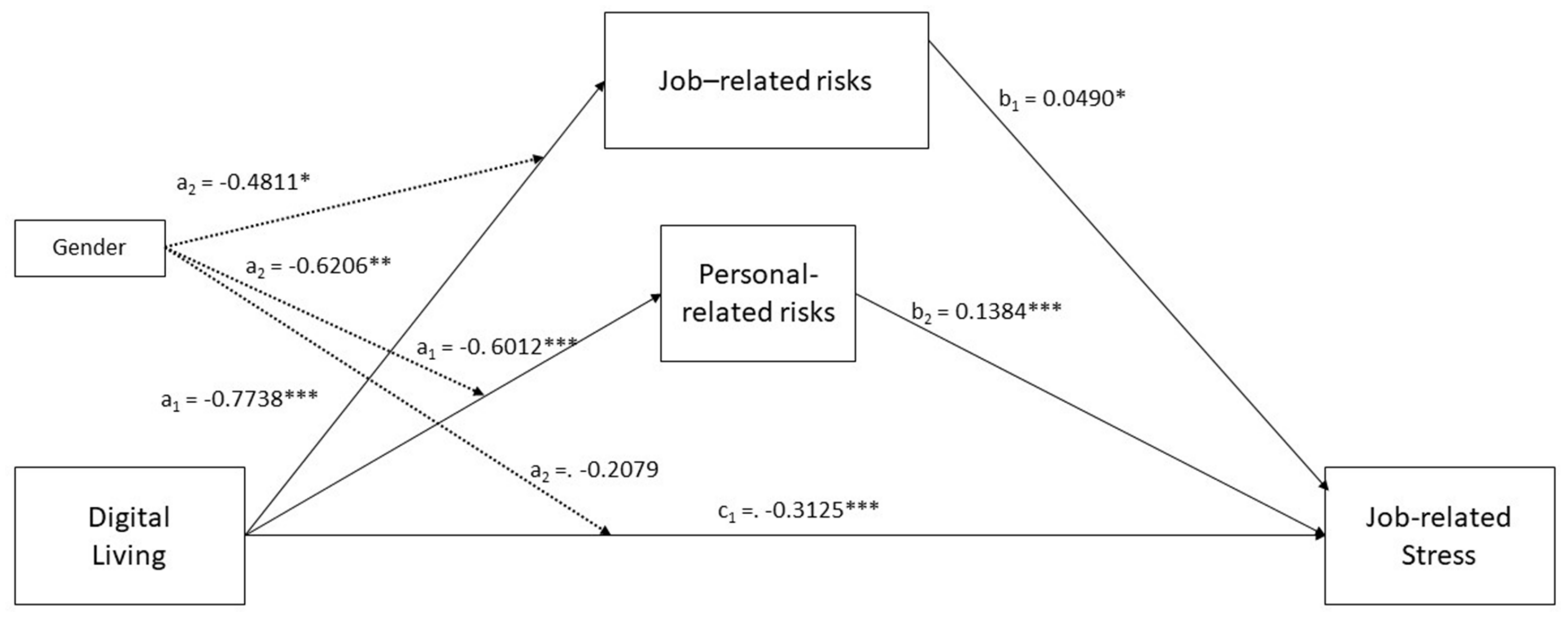
Unstandardized effects for the statistical model. Moderation effects are shown with black dotted lines. Gender assessed as 1 = males, 2 = females.

**Figure 4:**
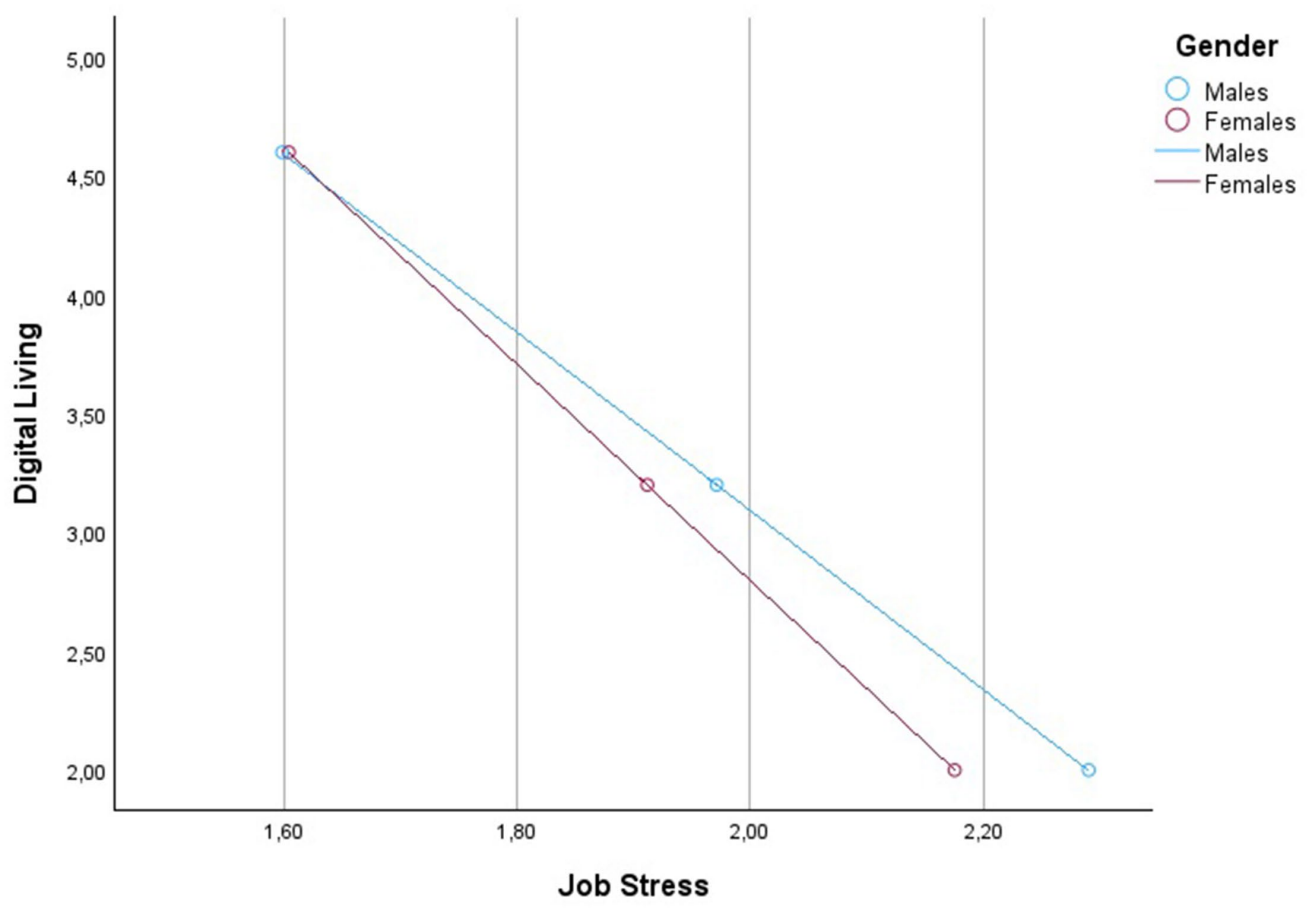
Graph of the interaction between Digital Living levels and Gender on Job Stress.

**Figure 5:**
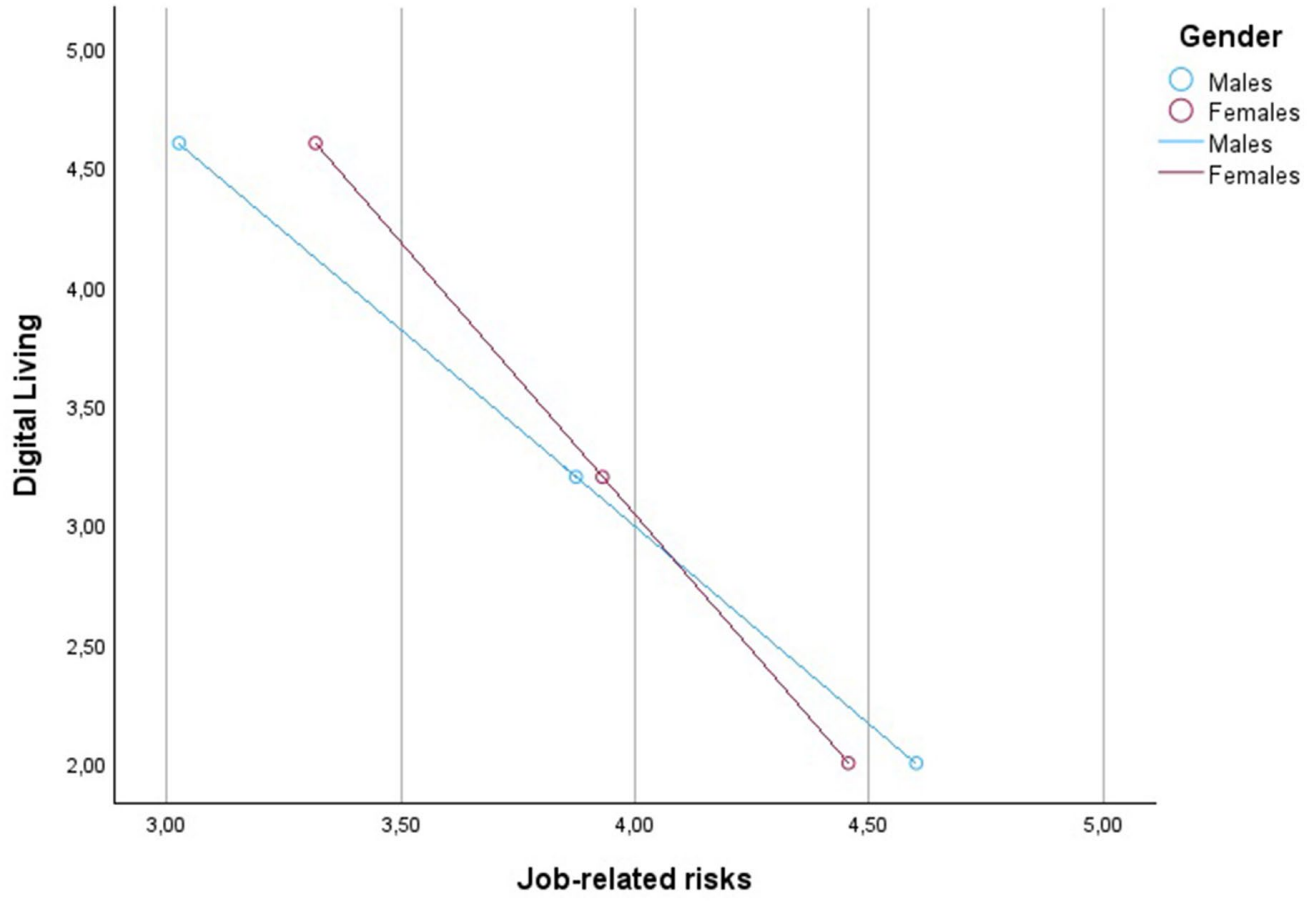
Graph of the interaction between Digital Living levels and Gender on Job-related risks.

**Figure 6:**
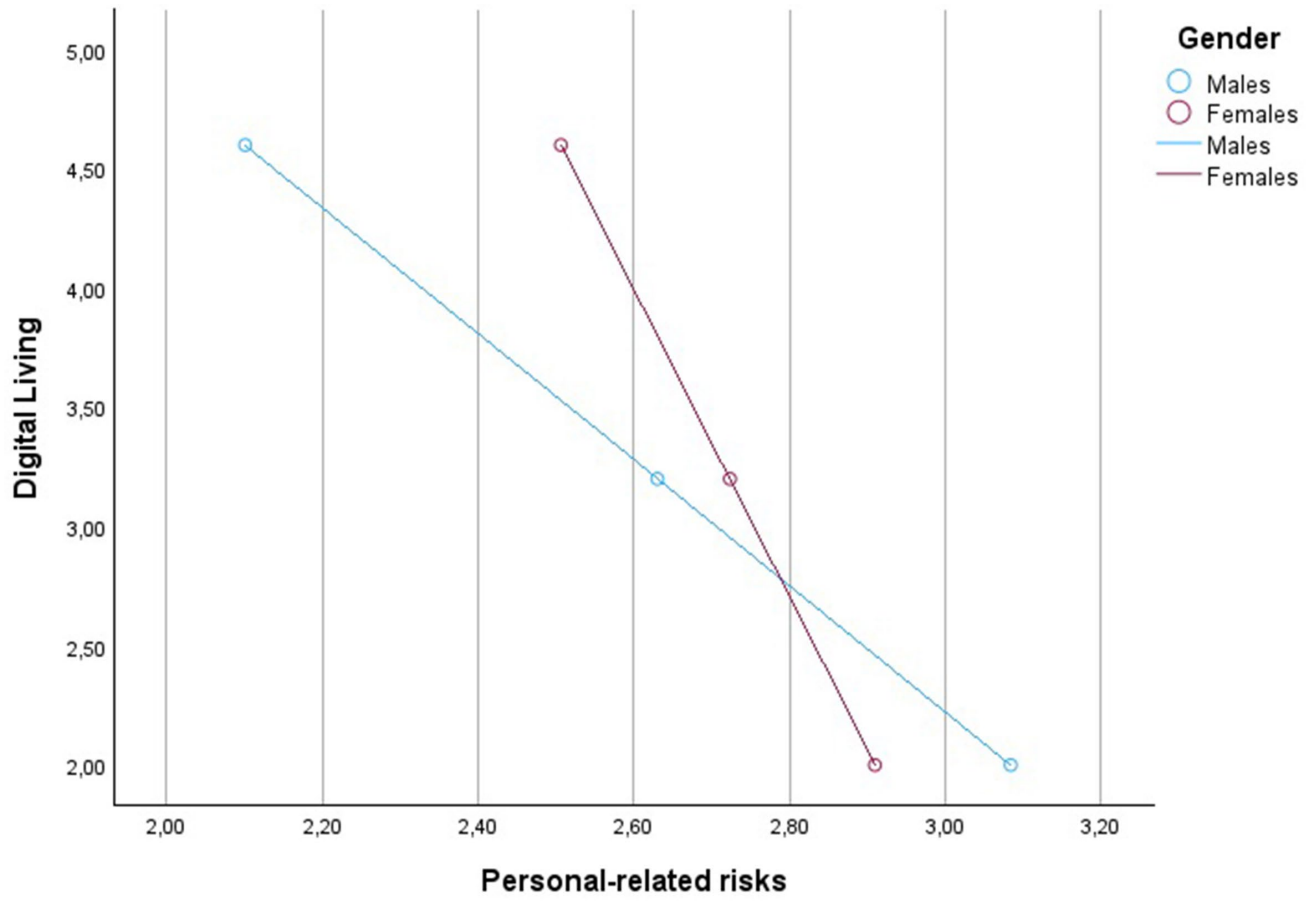
Graph of the interaction between Digital Living levels and Gender on Personal-related risks.

Detailed statistical values are shown in Table 2 and Figure 3. The graphical representation of the conditional effects is offered in Figures 4–6 and Tables 3–5.

**Table 2:** Effects of digital living on job-related risks moderated by gender.

Predictor	Coefficient	SE	*t*	*p*	95% CI
Constant	6.2936	0.4036	15.5934	< 0.001	[5.5013, 7.0859]
Digital living	−0.7738	0.1149	−6.7318	< 0.001	[−0.9994, −0.5481]
Gender	−0.4811	0.2377	−2.0243	0.0433	[−0.9477, −0.0146]
Digital living × Gender	0.1679	0.0694	2.4191	0.0158	[0.0317, 0.3042]

**Table 3:** Effects of digital living on personal-related risks moderated by gender.

Predictor	Coefficient	SE	*t*	*p*	95% CI
Constant	4.4607	0.3283	13.5868	<0.001	[3.8163, 5.1052]
Digital living	−0.6012	0.0935	−6.4304	<0.001	[−0.7848, −0.4177]
Gender	−0.6206	0.1933	−3.2101	0.0014	[−1.0001, −0.2411]
Digital living × Gender	0.2231	0.0565	3.9502	<0.001	[0.1122, 0.3339]

**Table 4:** Effects of digital living, job-related risks, and personal-related risks on job-related stress moderated by gender.

Predictor	Coefficient	SE	*t*	*p*	95% CI
Constant	2.4703	0.2994	8.2519	<0.001	[1.8827, 3.0580]
Digital living (T1)	−0.3125	0.0748	−4.1778	<0.001	[−0.4593, −0.1657]
Job-related risks (T2)	0.0490	0.0237	2.0722	0.0386	[0.0026, 0.0955]
Personal-related risks (T2)	0.1384	0.0291	4.7587	<0.001	[0.0813, 0.1955]
Gender	−0.2079	0.1498	−1.3882	0.1655	[−0.5019, 0.0861]
Digital living × Gender	0.0464	0.0439	1.0573	0.2907	[−0.0398, 0.1326]

**Table 5:** Conditional direct and indirect effects of digital living on job-related stress moderated by gender.

Direct effects					
Gender	Effect	SE	*t*	*p*	95% CI
Male	−0.2661	0.0363	−7.3397	<0.001	[−0.3372, −0.1949]
Female	−0.2197	0.0298	−7.3742	<0.001	[−0.2781, −0.1612]

Indirect effects via job-related risks			
Gender	Effect	BootSE	BootCI 95%
Male	−0.0297	0.0143	[−0.0587, −0.0029]
Female	−0.0215	0.0106	[−0.0429, −0.0021]

Indirect effects via personal-related risks			
Gender	Effect	BootSE	BootCI 95%
Male	−0.0523	0.0123	[−0.0773, −0.0285]
Female	−0.0215	0.0081	[−0.0393, −0.0075]

## Discussion

The findings of this study offer insights into the relationship between digital living and various aspects of occupational health, particularly in the context of job stress, job risk, and personal risk. These results have several implications for understanding the dynamics of digital living in the workplace and its impact on employees.

### Relationships of digital living on job stress and the role of mediators

The study corroborates Hypothesis 1 by establishing a significant, albeit less pronounced, relationship between digital living and job stress. This aligns with the growing body of research suggesting that digital engagement, while offering several benefits, can also contribute to occupational stress under certain conditions (Gadeyne et al., 2018). More importantly, the study supports Hypothesis 2 by demonstrating that both job risk and personal risk mediate this relationship. This finding is crucial as it suggests that the way digital living is related to job stress is not direct but rather occurs through its impact on perceived job and personal risks. This understanding agrees with previous findings, most of them reached under the COVID-19 pandemic and its increasing in teleworking (Ingusci et al., 2021). Some studies found that increased use of digital tools for remote work led to varied influences on job stress and productivity, with some experiencing increased stress due to information overload (Martin et al., 2022). But, at the same time, other studies noted that well-being measures, including job stress, were not adversely related for the majority of employees working remotely, suggesting a less direct relationship between digital living and job stress (Morikawa, 2020; Rubin et al., 2020). Among the mediating variables, recent empirical studies proposed the relationship of wok role overload and psychological detachment, as well as the negative spillover between work and family spheres as mediators between ICT use and different undesirable outcomes (Gadeyne et al., 2018; Elshaer et al., 2024). Following this avenue, recent studies explored the role of perceived risks in the intention to use ICT, showing promissory findings in different fields, as well as e-commerce (Zeng and Li, 2023), education (Baytar et al., 2023), social networking (Mýlek et al., 2023) and work.

### Gender as a moderator

The study’s results also validate Hypothesis 3, indicating that gender moderates the relationship between digital living, job risk, personal risk, and job stress. This is a significant contribution to the literature, highlighting the need to consider gender differences in occupational health research. The moderated mediation index’s statistical significance further underscores the importance of gender in understanding how digital living affects job and personal risks and, consequently, job stress. The significant interaction effects found in the study, particularly the stronger conditional negative effect of digital living on perceptions of job and personal risks for males, support Hypothesis 3.

The analysis reveals that digital living has a negative impact on job-related stress, but the magnitude of this effect is contingent on gender. Specifically, the conditional direct effects show that the negative relationship between digital living and job-related stress is more pronounced for males (−0.2661) compared to females (−0.2197). This suggests that as digital living increases, job-related stress decreases, but the strength of this effect is larger for men than women.

The study’s standpoint is that this gender difference highlights the importance of considering gender-specific interventions when addressing job-related stress in digital living contexts. The findings indicate that the pathways through which digital living influences job-related stress also differ by gender. The analysis of the indirect effects shows a significant, albeit small, moderated mediation effect through job-related risks. For males, the indirect effect is −0.0297, while for females it is −0.0215, indicating that job-related risks slightly mediate the relationship between digital living and job-related stress, and this mediation is stronger for men.

More importantly, the analysis reveals a more substantial indirect effect of digital living on job-related stress through personal-related risks, with the effect being notably stronger for males (−0.0523) than for females (−0.0215). This finding suggests that personal-related risks are a significant mediator in the relationship between digital living and job-related stress, particularly for males. In summary, the present study’s standpoint is that the impact of digital living on job-related stress is better predicted by considering the mediating roles of job-related and personal-related risks, and that these mediating processes are moderated by gender. The differences in conditional indirect effects across gender highlight how gender not only influences the direct relationship between digital living and job-related stress, but also shapes the pathways through which this relationship is manifested.

This suggests that men and women may experience and process digital living’s occupational risks differently. For men, higher digital living engagement seems to be associated with a more pronounced decrease in perceived job and personal risks, which might be attributed to varying coping mechanisms or differing experiences in digital environments, as other studies suggested (Carmassi et al., 2022; Rodríguez-Modroño, 2022).

These findings are in line with previous research on gender differences about risk perception (Gustafsod, 1998), and with females’ higher risk aversion (Harris and Jenkins, 2006). The present results agree with recent studies that continue stating the permanence of gender differences in risk perception and its wide range of applications (Jing et al., 2023; Măirean and Diaconu-Gherasim, 2023; Whitley and Bowers, 2023). Despite this consensus, contradictory findings have been obtained in specific contexts, as green consumption, with women showing less risk-aversion (Essiz et al., 2023). At a similar vein, other individual features have been highlighted as moderators of the perceived risk, as age, disabilities, low education or other disadvantaged conditions (Bermejo and Sánchez-Duarte, 2022; Chadwick, 2022; Köttl et al., 2022; Yang et al., 2023). For instance, recent studies suggested that telework implementation and teleworkers’ working conditions reproduce the structure of the labor market in traditional work. Those highly skilled workers, as regular workers and those employed in larger firms can participate in the telework more than non-regular workers or employees in small and medium size firms (Shin and Takenoshita, 2023). These findings indicate that perceptions of risk may be more significantly influenced by situational and contextual factors.

In this vein, recent experimental studies have emphasized that gender differences in the valuation of ambiguous options appear to result in lower risk aversion among males due to a more optimistic bias compared to females. Specifically, men tend to overweight favorable information and underweight unfavorable information when estimating the likelihood of a positive outcome. This suggests that both attitudinal and cognitive mechanisms may serve as subtle processes leading to higher risk aversion among women (Karmarkar, 2023). Despite the present findings supported the moderation hypothesis, the complexity of gender differences and their related variables should be taken into account. Some additional factors, as the gender gap in time use (Shin and Takenoshita, 2023), the gender differences in the stress-recovery (Suppakittpaisarn et al., 2023), and the influence of gender imbalance among specific professional sectors could contribute to alternative explanations for the findings (Khalid et al., 2020; Prasad et al., 2023).

In conclusion, this study contributes to a deeper understanding of how digital living impacts occupational health, following the line of recent studies (Sun et al., 2022). It highlights the complexity of these relationships, especially when considering mediating factors like job and personal risks and moderating factors like gender. These findings have practical implications for workplace policies and mental health strategies, emphasizing the need for gender-sensitive approaches in managing the impact of digital living on employee well-being. Moving forward, it would be beneficial to explore these dynamics in different occupational settings and across diverse cultural contexts to generalize these findings more broadly.

### Limitations and suggestions for future research

The limitations of this study are shaped by its methodological framework and the tools employed for data collection and analysis.

Firstly, the study’s participant pool consisted solely of Chinese ICT professionals. While this focus provides in-depth insights into this particular demographic, it limits the generalizability of the findings to other sectors or cultural contexts. The unique challenges and dynamics present in the Chinese ICT industry might not reflect the broader spectrum of industries or geographic regions. This distinction could affect the understanding of how digital living influences job stress and risks. Despite this, certain trends, such as the gendered digital divide, appear to be widespread (Picatoste et al., 2023).

Another limitation arises from the design of the instruments used. The Digital Living Scale, although comprehensive, primarily reflects the subjective experiences of individuals with digital technology in their social and daily lives. This subjective perspective may not capture the full spectrum of digital living’s impact, especially in a professional context, as recent meta-analysis suggested (Acilar and Sæbø, 2023). Moreover, the survey was conducted in English. But, despite that the participants were ICT workers; and their English languages skills are supposed high, a pilot test with a similar sample should be conducted to confirm that all the questions were fully understood. Similarly, the scales used to assess job-related and personal risks, adapted from previous studies, may not encompass all relevant dimensions of risk associated with digital living in the workplace. In the same vein, the conceptualization of gender as a binary option could preclude obtaining relevant information from all the participants that do not feel comfortable with this categorization. This option, that exclude those potential participants that categorize themselves as agender, bigender, genderfluid, demigender, genderqueer, or neutrois, would be associated to the selection bias.

Furthermore, the study’s reliance on self-reported data could introduce biases. Participants’ perceptions and responses might be influenced by their current mood, memory, or social desirability, which could skew the data. Additionally, the study’s design, involving three distinct data collection phases over a three-month period, aimed to mitigate common method bias. However, this approach might not entirely eliminate the potential for such bias, especially given the changing dynamics in the workplace over the course of the study.

The gradual decrease in participation over the three phases of the study also presents a limitation. This attrition could result in a non-response bias, where the views of those who dropped out might differ significantly from those who completed all phases.

Lastly, the statistical analysis, while robust, was based on Model 58 which, though well-suited for examining relationships between variables, may not capture the complexity of interactions in real-world settings. The use of gender as a moderating variable adds valuable insights, but future research could benefit from considering other demographic variables such as age, education level, and years of experience in the ICT sector. In a more sophisticated model, the relationships among digital use, risk perceptions, and job-related stress could be examined through a mediated chain that incorporates additional factors, such as work–family conflict and gendered time use (Chidambaram and Scheiner, 2023).

The findings of this study highlight the need for further research to explore the complex and gender-specific relationships between digital living, job-related risks, personal-related risks, and job-related stress. Future studies should seek to replicate and expand upon these results, investigating the generalizability of the observed gender differences across different organizational and cultural contexts. Additionally, research is warranted to identify the specific mechanisms and underlying processes that drive the differential impact of digital living on job-related stress for men and women. This could involve examining factors such as work-life balance, coping strategies, and gender-role expectations. Ultimately, a deeper understanding of these dynamics will enable the development of more targeted and effective interventions to mitigate job-related stress in the context of increased digital living. By addressing these avenues for future research, scholars can build upon the insights provided by this study and contribute to a more comprehensive understanding of the complex interplay between technology, gender, and workplace well-being.

In summary, while the study provides significant insights into the effects of digital living on job stress and risks in the Chinese ICT sector, these limitations highlight the need for cautious interpretation of the findings and suggest areas for future research to expand on these initial observations.

### Recommendations for human resource management interventions

The findings of this study provide valuable insights for Human Resource Management interventions in the context of digital living, job stress, job risk, and personal risk. These interventions can be tailored to address the specific dynamics and relationships uncovered in the research.

To address the relationship between digital living and job risk, HRM departments should consider developing and implementing comprehensive digital literacy and competency programs. These programs should focus not only on enhancing technical skills but also on integrating digital tools effectively into work processes (Ruparel et al., 2020). The goal is to minimize job risk associated with digital living, especially in environments where the use of digital technologies is prevalent. By improving employees’ digital competencies, organizations can leverage the benefits of digital living while reducing associated risks.

In terms of managing job stress, the study suggests that HRM should develop strategies that consider both job-related and personal risks as mediators (Xiao et al., 2020). This could involve creating support systems or resources that help employees manage the demands of digital living. For example, offering training on time management, privacy protection, and cybersecurity can help employees navigate the challenges posed by digital tools, thereby reducing job stress (Agrawal et al., 2024).

Additionally, the study’s findings on the moderating role of gender indicate the need for gender-sensitive approaches in HRM interventions. Since the impact of digital living on job and personal risk varies between males and females, HRM should tailor their interventions to address these differences. This could involve conducting gender-specific focus groups to understand better the unique challenges faced by each gender in digital environments and developing targeted strategies to address these challenges (Love et al., 2024).

Furthermore, HRM could also implement policies and practices that promote a balanced use of digital tools, considering that extensive digital engagement can lead to stress and risks. Encouraging regular breaks from digital devices, promoting face-to-face interactions, and creating ‘digital-free’ zones in the workplace could help mitigate the negative effects of prolonged digital engagement (McPhail et al., 2024).

The significant mediating roles of job-related risks and personal-related risks in the relationship between digital living and job-related stress have important implications for policymakers and organizational decision-makers. The findings suggest that interventions aimed at addressing job-related stress in the context of increased digital living should adopt a multifaceted approach that considers both the work-related and personal factors influencing this relationship. Policymakers should encourage the development of organizational policies and practices that help employees effectively manage the demands and challenges associated with digital living, such as providing training on time management, work-life balance, and coping strategies. Additionally, policies should promote a work culture that recognizes and addresses the unique personal-related risks that may arise from digital living, such as increased stress, burnout, and mental health concerns. Employers should be incentivized to implement comprehensive well-being programs that support employees’ physical, mental, and emotional needs. By addressing both the job-related and personal-related risks associated with digital living, policymakers can help organizations create healthier and more productive work environments that enable employees to thrive in the digital age.

Lastly, considering the significant impact of digital living on personal risks, HRM should also focus on employees’ overall well-being. This could include initiatives like wellness programs, mental health support, and stress management workshops. Such initiatives would not only address the direct effects of digital living on personal risk but also contribute to a healthier (Fernández-Salinero et al., 2023), more productive work environment.

The design and implementation of such interventions would be affected by challenges and difficulties both related to the organizations and the individuals. For instance, if organizations are in fact interested in the reduction of the gender gap, and the potential use of telework to reduces gender inequalities in the labor market, the implementation of key performance indicators (KPIs) to evaluate the success of the HRM intervention would contribute to an objective assessment of advancements. Related to the individuals, cultural factors can exert a complex influence that impact on the effectiveness of the suggested interventions.

In conclusion, the study’s findings offer a roadmap for HRM to develop targeted, effective interventions that address the complexities of digital living in the workplace. By focusing on digital literacy, stress management, gender-sensitive approaches, balanced digital engagement, and overall well-being, HRM can play a crucial role in fostering a positive work environment in the digital age.

## Conclusion

This present study confirms that the impact of digital living on job stress is indirect, mediated by perceptions of risk, and moderated by gender differences. These findings not only align with existing literature on the increased teleworking during the COVID-19 pandemic but also extend our understanding by identifying gender as a significant factor in how digital technologies affect occupational stress. The study suggests that gender-sensitive approaches in workplace policies and mental health strategies are essential to address the nuanced impacts of digital living on employee well-being.

Furthermore, the research underscores the importance of considering individual differences, such as gender, in assessing and managing the occupational health implications of digital living. The differential impact observed across genders calls for targeted interventions that accommodate the varying experiences and perceptions of risk associated with digital engagement in the workplace. Despite the existence of certain limitations, that could impact the generalization of the present findings, the study provides a foundation for future research aimed at optimizing the integration of digital technologies in professional settings, ensuring that workplace practices support the health and productivity of all employees, irrespective of gender.

## Data availability statement

The raw data supporting the conclusions of this article will be made available by the authors, without undue reservation.

## Ethics statement

The studies involving humans were approved by The Ethics Committee of City University. The studies were conducted in accordance with the local legislation and institutional requirements. The participants provided their written informed consent to participate in this study.

## Author contributions

SH: Writing – review & editing, Writing – original draft.
